# Immunohistochemical Study of Antioxidant Enzymes Regulated by Nrf2 in the Models of Epileptic Seizures (KA and PTZ)

**DOI:** 10.1155/2019/1327986

**Published:** 2019-03-24

**Authors:** María Fernanda Munguía-Martínez, Concepción Nava-Ruíz, Amairani Ruíz-Díaz, Araceli Díaz-Ruíz, Petra Yescas-Gómez, Marisela Méndez-Armenta

**Affiliations:** ^1^Laboratório de Neuropatologia Experimental, Instituto Nacional de Neurología y Neurocirugía, Manuel Velasco Suárez, Mexico; ^2^Departamento de Neuroquímica, Instituto Nacional de Neurología y Neurocirugía, Manuel Velasco Suárez, Mexico; ^3^Departamento de Neurogenética, Instituto Nacional de Neurología y Neurocirugía, Manuel Velasco Suárez, Mexico

## Abstract

Epilepsy is a neurological disorder characterized by recurrent spontaneous seizures due to an imbalance between cerebral excitability and inhibition, with a tendency towards uncontrolled excitability. Epilepsy has been associated with oxidative and nitrosative stress due to prolonged neuronal hyperexcitation and loss neurons during seizures. The experimental animal models report level of ATP diminished and increase in lipid peroxidation, catalase, and glutathione altered activity in the brain. We studied the immunohistochemical expression and localization of antioxidant enzymes GPx, SOD, and CAT in the rat brains treated with KA and PTZ. A significant decrease was observed in the number of immunoreactive cells to GPx, without significant changes for SOD and CAT in KA-treated rats, and decrease in the number of immunoreactive cells to SOD, without significant changes for GPx and only CAT in PTZ-treated rats. Evident immunoreactivity of GPx, SOD, and CAT was observed mainly in astrocytes and neurons of the hippocampal brain region in rats exposed at KA; similar results were observed in rats treated with PTZ at the first hours. These results provide evidence supporting the role of activation of the Nrf2 antioxidant system pathway against oxidative stress effects in the experimental models of epileptic seizures.

## 1. Introduction

Epilepsy is one of the most common neurologic disorders that affects over 50 million people worldwide; it has an incidence of approximately 50 new cases per year per 100,000 population; approximately 75% of epilepsy begins during childhood, reflecting the heightened susceptibility and high societal cost [1, 2]. Epilepsy is characterized by recurrent spontaneous convulsive or nonconvulsive seizures due to an imbalance between cerebral excitability and inhibition, with a tendency towards uncontrolled excitability [3]. Epilepsy can be divided mainly into two types: (1) mesial temporal lobe epilepsy which involves the hippocampus, parahippocampal gyrus, and amygdala and (2) lateral lobe epilepsy which involves the neocortex, which is the most prominent and common of acquired epilepsies [4, 5]. It has been widely recognized in the physiopathology of epilepsy that oxidative stress has a fundamental role within the mechanisms of action of the disease; there is evidence that neuronal hyperexcitability and oxidative injury produced by an excessive production of free radicals may play a role in the initiation and progression of epilepsy [6].

Oxidative and nitrosative stress results from an imbalance between the production of reactive oxygen/reactive nitrogen species (ROS/RNS) and the capacity of endogenous antioxidant system to remove free radical production [7]. ROS/RNS are chemically reactive and are formed as a natural by-product of the normal metabolism. The brain is particularly susceptible to oxidative stress being the most aerobically active organ in the body due to its high metabolic demands [8]. The brain is generally in a redox balance between oxidative and reactive conditions; however, ROS/RNS have damaging effects when they are produced in excessive amounts which generate large numbers of potential harmful intermediates that cause cellular dysfunction [8]. The most important antioxidant enzymes include superoxide dismutase (SOD), glutathione reductase, glutathione peroxidase (GPx), and catalase (CAT); SOD enzymes, including CuZnSOD and MnSOD, facilitate dismutation of superoxide radicals to generate H_2_O_2_, which is further removed by CAT and glutathione peroxidase enzymes [9], and the antioxidants play an important role in cellular defense against ROS/RNS. The antioxidants can scavenge ROS/RNS or increase the ability to neutralize ROS/RNS by inducing the expression of genes involved in the cellular protection [10], so the induction of these cytoprotective enzymes in response to oxidative stress is a priority activity. The Nrf2 factor regulates the expression of around 250 genes and is found in the cytoplasm bound to the repressor protein Keap 1 which under normal conditions is eliminated by the ubiquitin pathway, whereas that when it is activated by external stimuli, the Nrf2 factor translocates to the nucleus which binds to the antioxidant response element (ARE) in the promoter region of the gene initiating the transcription of numerous cytoprotective enzymes. The Nrf2 factor responds to oxidative stress conditions; the increment in the transcription of the antioxidant enzymes can serve as indicators of cellular mechanisms that act in repair processes or cellular protection against stressful stimuli [11, 12]. The genes that predominantly regulated by Nrf2 include heme oxygenase-1 (HO-1), NAD(P)H, quinone oxidoreductase 1 (NQO1), glutathione peroxidase, superoxide dismutase, and catalase between others [13]. Some authors have reported the neuroprotective role that the NRf2 factor could play in epilepsy; when an increase in cellular oxidative stress is generated due to cellular overexcitation, the protection mechanisms are increased by the induction of cytoprotective enzymes mediated by this factor, in astrocytes and neurons in culture increased levels of Nrf2 as well as some antioxidant enzymes that have been observed [13, 14]. The activation of Nrf2 has been also reported in the hippocampal tissue from patients and mice with temporal lobe epilepsy [15].

The experimental epilepsy models have been developed to assess the pathophysiology of epileptic seizures and have played a fundamental role in our better understanding of the molecular mechanisms associated with seizure development. The experimental animal models can be experimental seizures induced by chemical convulsants such as kainic acid (KA) or pentylenetetrazol (PTZ) [16, 17]. KA is a glutamatergic agonist that produces a series of behavioral signs culminating in status epilepticus (SE) with neuropathological changes that are similar to human epilepsy [18], whereas that the model of PTZ induces convulsive seizures by imbalance between neural inhibition and excitation when interacting with the GABAergic system, with repeated injection of subconvulsive dose of PTZ causes gradual development of seizure culminating to generalized tonic-clonic seizures [19]. The aim of this paper was to examine through immunohistochemical study the localization and expression of antioxidant enzyme system (GPx, SOD, and CAT) in the hippocampal regions of the rat brains treated with KA and PTZ.

## 2. Material and Methods

### 2.1. Animals

The experiments were performed on adult male Wistar rats, NIH bred in-house strain, weighing 200-220 g, randomly distributed to three different groups. They were housed under standard conditions, fed a standard chow diet (Purina chow), and had free access to water. Room darkness was maintained between 19:00 h and 7:00 h, room temperature at 25°C, and relative humidity at 40%. The animals were handled according to the National Institutes of Health (USA) *Guide for the Care and Use of Laboratory Animals*, and this protocol was approved by the Bioethics Committee of the National Institute of Neurology and Neurosurgery of Mexico.

### 2.2. Experimental Procedure

The animals (*n* = 6) per group were injected i.p. with saline solution (the control group) which were sacrificed immediately after injection to serve as time 0 control. Other group of animals (*n* = 6) was injected with single doses of 10 mg/kg KA [17]. Animals are treated with KA developing “have staring” spells; head nodding and several wet dog shakes during the first 20-30 min in the final phase present a recurrent limbic motor seizure evolving to a full motor limbic SE. The animals with extensive tonic-clonic seizures were included in the time course study and were sacrificed at 1, 6, 12, 24, and 48 h. For PTZ kindling, the animals were treated with a subconvulsant 3 doses of 25 mg/kg (body weight) of PTZ for i.p. each for 15 minutes and were sacrificed at 1, 12, 24, 48, and 72 h after the last injection [18]. The PTZ injections induced generalized clonic or tonic seizures consistent with Racine scale, whose classification is as follows: 0—no reaction; 1—stereotypic mounting, eye blinking, and/or mild facial clonus; 2—head nodding and/or multiple facial clonus; 3—myoclonic jerks in the forelimbs; 4—clonic convulsions in the forelimbs with rearing; and 5—generalized clonic convulsions and loss of balance. The latency and duration of seizure were observed behaviorally [19, 20].

### 2.3. Histopathological and Immunohistochemical Study

The animals were deeply anesthetized with overdoses of pentobarbital (100 mg/kg i.p.) and perfused via the ascending aorta with phosphate-buffered saline (PBS), followed by 10% *w*/*v* buffered formalin solution at 4°C for histopathological examination. The whole brains were removed from the skull, were immersed in the same fixative, and after were cut with a matrix (coronal rodent brain matrix, EMS) at 2 mm thick [21]. The sections between −3.14 and −4.16 mm posterior to the bregma suture containing the dorsal hippocampus were selected using a Paxinos and Watson stereotaxic atlas [22]. Brain tissue samples were embedded in paraffin, were cut at 5 *μ*, and stained with cresyl violet. Pathological analysis was performed for all rats with a light microscope Axio Lab A1 Zeiss with Axiocam ICC5 camera at a magnification of 1000x.

For immunohistochemical studies, a LSAB System HRP (Dako, Carpinteria, CA), antisuperoxide dismutase polyclonal antibody (Abcam International, USA), antiglutathione peroxidase (Abcam International, USA), and catalase (Abcam International, USA) were used. In brief, according to Juárez-Rebollar et al. [21], the sections were deparaffinized, after hydrated with decreasing alcohol concentrations and washed three times for 3 min each time in 0.01 M phosphate-buffered saline (PBS, pH 7.4) for heat-induced epitope retrieval; the sections were boiled in citrate buffer (pH 6 or 9) in a microwave oven for 2 × 10 min. The sections were preincubated with 0.3% hydrogen peroxide in PBS and later incubated with SOD antibody (1 : 100), GPx (1 : 100), and CAT (1 : 100) by 90 min at room temperature. Slices were washed two times with PBS for 2 min followed by incubation with a secondary biotinylated antisera and then immersed in avidin–biotin peroxidase complex (LSAB System HRP, Dako, Carpinteria, CA) for 20 min at room temperature. The negative control sections were treated in the same way as described above except for each primary antibody was omitted. The immune reaction resulted in the oxidation of the 3,3′-diaminobenzidine by peroxidase (Liquid DAB, Dako, Carpinteria, CA) into an insoluble brown precipitate. Counterstaining with hematoxylin was performed after immunostaining. For immunostainings, changes in the number of these immunopositive cells were counted under a light microscope Axio Lab A1 Zeiss with Axiocam ICC5 camera at a magnification of ×1000.

### 2.4. Morphometric Analysis

Three slides per rat were prepared and analyzed under light microscopy. Images from ten immersion oil fields were selected of the hippocampus area. Normal and damaged cells were counted in those images. The damaged cells were identified according to the criteria followed by Chang [23]. Percentages of damaged cells were obtained by dividing the number of damaged cells between total cells in the ten fields counted in each slide, and the total number of cells was expressed as the average of cells per field in each slide per rat. Similar analysis was performed to evaluate the immunohistochemical positive cells.

### 2.5. Statistical Analysis

An exploratory analysis of the data was performed to determine normal distribution (Kolmogorov-Smirnov test) and homogeneity of variances, applying the Levene test. The results of positive cells for GPx and CAT were analyzed with one-way ANOVA, followed by the Dunnett test for multiple comparisons. The values of the altered cells and positive cells for SOD were examined by the Kruskal-Wallis test, followed by the Mann-Whitney *U* test (due to lack of normal distribution and homogeneity of variances of data). All analyses were performed with an SPSS 22.0 software. Differences were considered statistically significant when *p* < 0.05.

## 3. Results

### 3.1. Behavioral Observations

The animals treated with KA and PTZ shown high scores of spontaneous seizures with hyperexcitability (SE period), with progressive behavioral characterized by hypersalivation, immobility followed appearance of the mild forelimb and facial clonus, masticatory movements head nodding and wet dog shakes, and finally tonic-clonic seizures involving all the four limbs (data not shown).

### 3.2. Histological and Immunohistochemical Analysis

**Figure 1:**
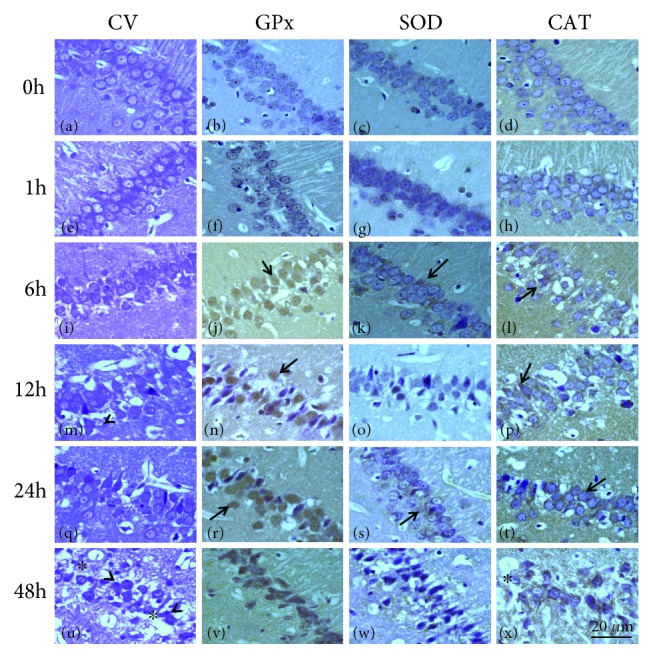
Representative micrographs of the hippocampal fields of rats treated with KA. Area in CA1 in the control hippocampus (a–d), 1 h group (e–h), 6 h group (i–l), 12 h group (m–p), 24 h group (q–t), and 48 h group (u–x). The sections stained with cresyl violet, showing neuronal cells with karyolysis (^), pyknotic nuclei, interstitial edema, and cellular necrosis (^∗^) (i, m, q, and u). A GPx-, SOD-, and CAT-positive staining of CA1 and CA3 neurons can be observed. The hippocampal tissues increased GPx, SOD, and CAT immunoreactivity mainly in neurons and astrocyte with nuclei strongly stained in the experimental groups (↑). Preparations were stained for cresyl violet (a, e, i, m, q, and u) and immunostained for GPx (b, f, j, n, r, and v), SOD (c, g, k, o, s, and w), and CAT (d, h, l, p, t, and x) were observed. Magnification: 1000x. Scale bar: 20 *μ*.

**Figure 2:**
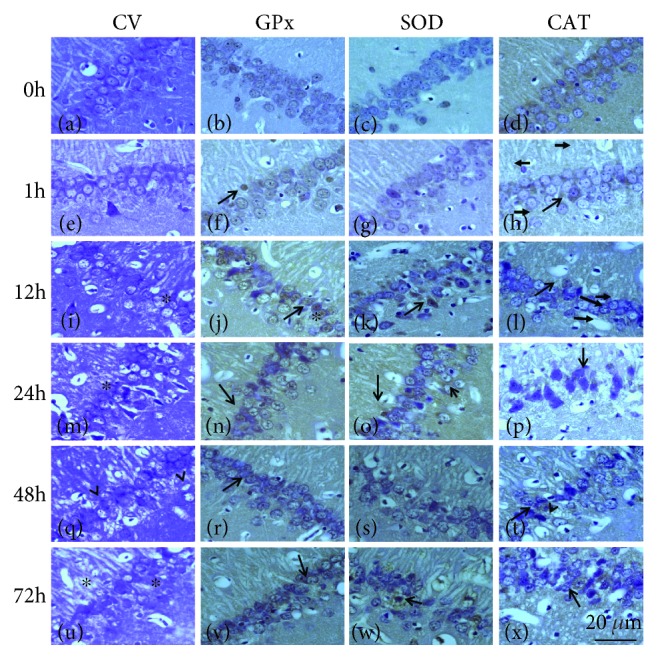
Representative micrographs of the hippocampal fields of rats treated with PTZ. Area in CA1 in the control hippocampus (a–d), 1 h group (e–h), 12 h group (i–l), 24 h group (m–p), 48 h group (q–t), and 72 h group (u–x). The sections stained with cresyl violet, showing neuronal cells with karyolysis (^), pyknotic nuclei, and slight interstitial edema (^∗^) with loss cells (i, m, q, and u). GPx-, SOD-, and CAT-positive cells are seen in neuron and several astrocytes. The hippocampal tissues increased GPx, SOD, and CAT immunoreactivity mainly in neurons and astrocyte with nuclei strongly stained in experimental groups (↑). Preparations were stained for cresyl violet (a, e, i, m, q, and u) and immunostained for GPx (b, f, j, n, r, and v), SOD (c, g, k, o, s, and w), and CAT (d, h, l, p, t, and x) were observed. Magnification: 1000x. Scale bar: 20 *μ*.

The histopathological analysis shows the pyramidal cells with the nucleus, nucleus and cytoplasm, and glial cells and a neuropil with a normal appearance in the hippocampal region (Figures 1(a)–1(d) and 2(a)–2(d)). In the group treated with KA, no evident cell damage was observed at 1 h (Figure 1(e)); while at 6 and 12 h, the damage to the pyramidal cells is observed with an increase with the presence of hyperchromatic nuclei, loss of pyramidal cells, and interstitial edema (Figures 1(i) and 1(m)). At 24 and 48 h, the damage is observed as severe damage in the pyramidal cells of the hippocampus; there are numerous pyknotic cells, cellular atrophy, loss of cells, presence of intense interstitial edema, and neuronal necrosis in the hippocampal subfields CA1 and CA3 (Figures 1(q) and 1(u)). On the other hand, in the group treated with PTZ, no damage was observed in the pyramidal cells at 1 h of treatment (Figure 2(e)). At 12 and 24 h, the damage was evident of nuclear changes, presence of pyknotic pyramidal cells, and numerous nuclei with karyolysis (Figures 2(i) and 2(m)); at 48 and 72 h (Figures 2(q) and 2(u)), there is an increase in the loss of pyramidal cells, severe karyolysis with lack of nuclei, and intense interstitial edema.

Except for minor differences, the general pattern of immunoreactivity with all three antibodies was similar in the control group; an evident immunoreactivity for GPx was present mainly in the CA1-CA3 neurons (Figures 1(b) and 2(b)). Rats treated with AK at 1 h show a slight increase in the GPx immunoreactivity of the cells (Figure 1(f)), while a strong immunoreactivity was observed in the pyramidal cells of the rats at 6, 12, 24, and 48 h, although several cells showed no immunoreactivity due to cell damage present (Figures 1(j), 1(n), 1(r), and 1(v)). The SOD immunoreactivity was slight in rats of the control group (Figures 1(c) and 2(c)), whereas that a higher density of immunoreactivity was observed in the cytoplasm of neurons and glial cells at 1 h and 6 h (Figures 1(g) and 1(k)), with a slight increase of intensity at 12 h after treatment with AK (Figure 1(o)); the SOD immunoreactivity was intense in some cells at 24 and 48 h (Figures 1(s) and 1(w)). Immunostaining with anti-CAT antibody shows slight reactivity in rats of 1 and 6 h in neurons and glial cells (Figures 1(h) and 1(l)); the CAT immunoreactivity in CA3-CA1 neurons appears to be stronger at 12 and 24 h (Figures 1(p) and 1(t)) after KA administration in spite of a decrease in the number of cell. On the other hand, rats treated with PTZ showed a weak immunoreactivity in the neurons and glial cells of CA3-CA1 for GPx, SOD, and CAT at 1 h (Figures 2(f)–2(h)), with an increase in immunostaining at 12 and 48 h was observed (Figures 2(j)–2(p)); all three antibodies were also present in all neural layers at 72 h (Figures 2(v)–2(x)) and could not be detected in areas showing complete neuronal loss. In a semiquantitative attempt to summarize the above data, we have listed the major centers that show distinct intense immunoreactivity on a scale of +/+++ (Table 1).

### 3.3. Morphometric Evaluation

**Figure 3:**
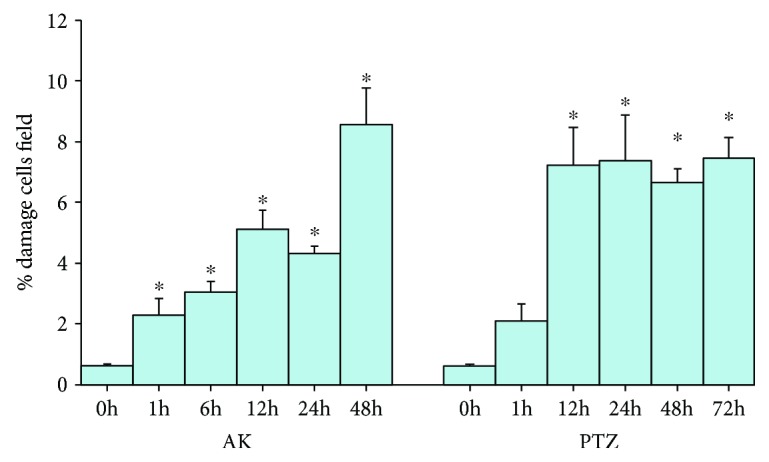
Percentage of cellular damage in the hippocampal region of rats exposed to KA and PTZ. Values are means ± S.E.M. expressed as the percentage of damage cells counted in ten fields per area in three slides per rat. ^∗^*p* < 0.05 vs. control.

The average of damaged cells in the rats of the control group was 0.6 ± 0.07, while in the rats treated with KA 2.28 ± 0.53 at 1 h, 3.03 ± 0.37 at 6 h, 5.13 ± 0.59 at 12 h, 4.3 ± 0.23 at 24 h, and 8.56 ± 1.2 at 48 h, significant changes were observed at all the times analyzed. On the other hand, the average of damaged cells in rats treated with PTZ was 0.6 ± 0.07 at 1 h (2.1 ± 0.53), 12 h (7.2 ± 1.25), 24 h (7.3 ± 1.48), 48 h (6.65 ± 0.46), and 72 h (7.4 ± 0.67) resulting in a significant increase in damaged cells in the time studied (Figure 3).

**Figure 4:**
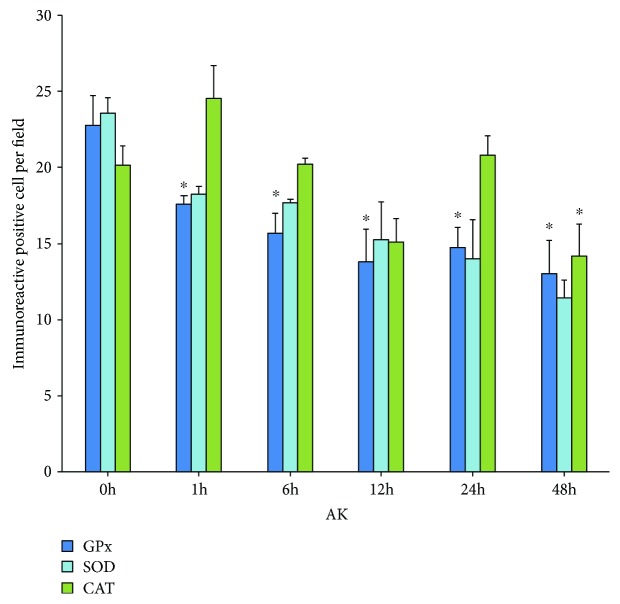
Number of immunoreactive cells to GPx, SOD, and CAT in group treated with KA. Values are means ± S.E.M. expressed as the number of immunoreactive cells counted in ten fields per area in three slides per rat. ^∗^*p* < 0.05 vs. control.

A significant lower number of immunoreactive GPx cells was found in the rats treated with KA, and all the times were studied at 0 h (22.7 ± 1.93), 1 h (17.6 ± 0.55), 6 h (15.7 ± 1.3), 12 h (13.8 ± 2.14), 24 h (14.7 ± 1.29), and 48 h (13.0 ± 2.17); also significant change was observed in cells immunoreactive to CAT at 48 h with respect to control (0 h, 20.1 ± 1.24; 1 h, 24.5 ± 2.15; 6 h, 20.2 ± 0.4; 12 h, 15.1 ± 1.55; 24 h, 20.8 ± 1.27; and 48 h, 14.1 ± 2.13) while no significant changes in the SOD immunoreactivity at 0 h (23.5 ± 1.06), 1 h (18.2 ± 0.52), 6 h (17.7 ± 0.2), 12 h (15.2 ± 2), 24 h (14 ± 2.55), and 48 h (11.42 ± 1.2); however, it observed a tendency to decrease (Figure 4).

**Figure 5:**
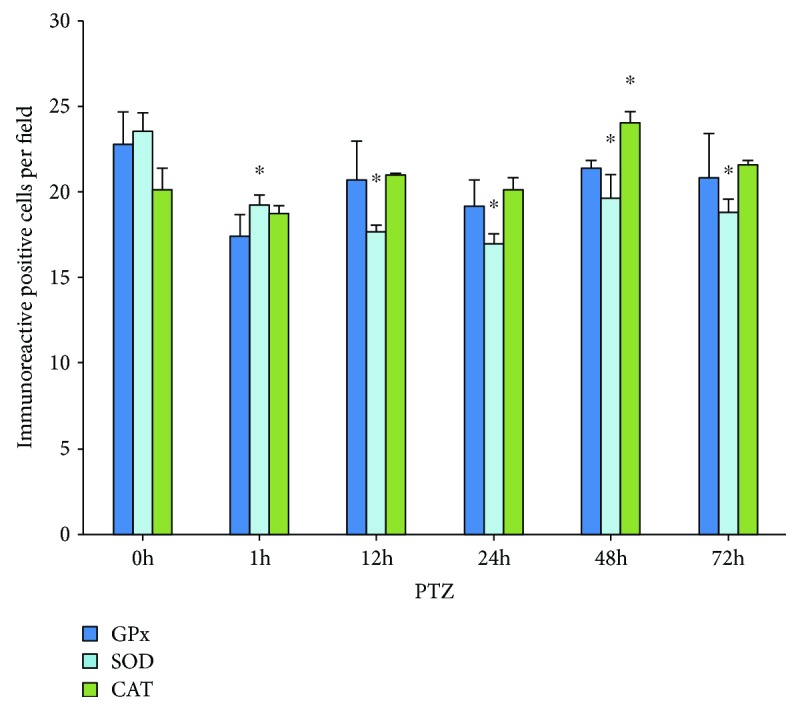
Number of immunoreactive cells to GPx, SOD, and CAT in group treated with PTZ. Values are means ± S.E.M. expressed as the number of immunoreactive cells counted in ten fields per area in three slides per rat. ^∗^*p* < 0.05 vs. control.

A similar quantification was performed in the group treated with PTZ for the cells immunoreactive to GPx, SOD, and CAT. The results showed significant changes with respect to the control group for SOD (0 h, 23.5 ± 1.06; 1 h, 19.2 ± 0.52; 12 h, 17.6 ± 0.37; 24 h, 17 ± 0.56; 48 h, 19.6 ± 1.39; and 72 h, 18.8 ± 0.77) and CAT (0 h, 20.15 ± 1.24; 1 h, 18.73 ± 0.43; 12 h, 21 ± 0.05; 24 h, 20.1 ± 0.75; 48 h, 24.±0.6; 72 h, 21.6 ± 0.23). GPx (0 h, 22.7 ± 1.93; 1 h, 17.4 ± 1.24; 12 h, 20.7 ± 2.29; 24 h, 19.2 ± 1.51; 48 h, 21.3 ± 0.49; and 72 h, 20.8 ± 2.61) showed no changes in the times analyzed (Figure 5).

## 4. Discussion and Conclusion

In this paper, we show, through an immunohistochemical study, the localization and expression of antioxidant enzymes (GPx, SOD, and CAT) in two experimental models of epileptic seizures. Many experimental reports have demonstrated the involvement of oxidative stress in seizures associated with brain damage and the molecular mechanism that contributes to the pathophysiology of epilepsy [8, 17, 24]. Oxidative stress is the condition that occurs when the balance between prooxidants and oxidants moves towards prooxidants causing disturbances at the cellular level inducing an increase in organic damage [7, 24]. The overproduction of ROS/RNS generating increased levels of oxidative stress contributes to neuronal hyperexcitability and neuronal cell death through numerous mechanisms which include damage to membrane proteins, such as neurotransmitter receptors and neurotransmitters and ionic channels [5, 8]. It is known that the experimental models of epileptic seizures induced by AK and PTZ generate an increase in oxidative stress causing cellular damage [6, 16]. The pyramidal neurons of the hippocampus are particularly sensitive to the effects of AK because it acts by overacting the ionotropic receptors of glutamate producing an exictotoxic effect and cell death selectively in the CA3 and CA1 subfields and dentate gyrus of the hippocampus [16], and a series of repeated injections of PTZ (which blocks the GABA receptors involved in the control of neuronal excitability) is required to induce an effect that causes the loss of cells in the hippocampus in experimental animals [25]. In this work, we show an increase in the number of damaged cells (Figure 3) with karyolysis, cellular atrophy, and neural necrosis, in the hippocampal region of the rats treated with KA and PTZ, which agrees with what has been reported about these epileptic seizure models [17, 24, 26].

In the brain, there are protective mechanisms against the formation of reactive oxygen and nitrogen species, in response cells which increase their antioxidant defenses through the activation of the nuclear factor erythroid 2-related factor (Nrf2) [13, 15, 27]; Nrf2 is a key regulator in redox balance and intracellular signaling by increasing the expression of various endogenous antioxidant enzymes by binding to antioxidant response elements (AREs). The regulated ARE genes are preferentially activated in astrocytes, which consequently have a more efficient detoxification and antioxidant defenses better than neurons so they protect them against oxidative stress [27]; in animals treated with KA and PTZ, we have observed an increase in the expression of Nrf2 (*data in press*); moreover, the presence of an evident increase in immunoreactivity to enzymes regulated by this factor suggests its activation in the epileptic seizure models.

The antioxidant enzyme system regulated by Nrf2 mainly includes SOD (CuZnSOD and MnSOD) which is found in the mitochondria and in the cytosol, facilitating the dismutation of superoxide radicals to generate H_2_O_2_ [8]; the H_2_O_2_ can be neutralized in H_2_O through GPx in the cytosol and can also be eliminated by CAT in the mitochondria and peroxisomes [9]; in this paper, we observed a decrease in the immunoexpression of some of these enzymes in both experimental models. Studies in animals treated with KA and pilocarpine have shown an increase in the activity of CAT, SOD, and GPx [26, 28, 29]. Similar results have also been reported in patients with epilepsy, finding increase or decrease in the activity of SOD, CAT, and GPx, as well as other indicators of oxidative stress such as lipoperoxidation [30–32]; decrease in GPx levels and elevated CAT levels in neocortex tissue from patients with epilepsy has also been reported [33]. On the other hand, histopathological studies with immunofluorescence have shown the distribution of these enzymes in the hippocampus of patients with epilepsy, reporting that CAT and SOD were located mainly in neurons, whereas GPx was observed in capillaries [29]. Also, CAT has been shown in neurons and glia [34], likewise glutathione in reactive neurons and astrocytes in rats treated with KA [35]. According to these studies, we observed a similar distribution of GPx, SOD, and CAT immunoreactivity in CA1 and CA3 neurons and glia of the hippocampus in rats treated with KA and PTZ.

The morphometric analysis showed a significant increase in the percentage of damaged cells in both KA and PTZ groups (Figure 3). In KA group, a significant decrease in the number of immunoreactive cells for the GPx was observed, while that SOD keeps unchanged and CAT only diminishes at 48 hours (Figure 4); however, the intensity in the immunoreactivity was enhanced and maintained in the cells that were analyzed (Table 1). Similar results were observed in rats treated with PTZ, and an increase in neuronal loss at 12, 24, 48, and 72 h could be observed (Figure 3); moreover, significant differences with a decrease in the number of immunoreactive cells to SOD and only increase for SOD at 48 h in animals of this group were found (Figure 5). These results show a decrease of immunoreactive cells of GPx which is probably affected by KA, while that SOD and CAT the immunoexpression remains without significant changes for more time, suggesting that SOD and CAT probably play a more important role so they can be considered the main antioxidant enzymes in these experimental models of epileptic seizures. These results agree with that reported by other authors showing increased levels of SOD and CAT and decreased GPx in tissue from epileptic patients [30, 33], as well as experimental rodent model epilepsy [26].

In conclusion, our data indicate changes in the immunoexpression of antioxidant enzymes in neurons and glia in the experimental models of epileptic seizures, which agree with that reported by other authors providing evidence that support the role played by the antioxidant system pathway the activation of factor Nrf2 against oxidative stress as a relevant defense mechanism in pathological conditions derived from the epileptic seizures.

## Figures and Tables

**Table 1:** Results of the semiquantitative immunohistochemical evaluation of GPx, SOD, and CAT according to the KA or PTZ group.

Treatment	Time	GPx	SOD	CAT
KA	0 h	+	+	+
	1 h	+	++	++
	6 h	++	++	++
	12 h	++	+	++
	24 h	++	++	+++
	48 h	++	++	++

PTZ	0 h	+	++	+
	1 h	+	+	+
	12 h	+++	++	++
	24 h	++	++	++
	48 h	++	++	+++
	72 h	++	++	+++

Immunoreactivity: +: slight; ++: moderate; +++: intense.

## Data Availability

The pathological and immunohistochemical data used to support the findings of this study are available with the corresponding author upon request.
